# Time-varying information measures: an adaptive estimation of information storage with application to brain-heart interactions

**DOI:** 10.3389/fnetp.2023.1242505

**Published:** 2023-10-18

**Authors:** Yuri Antonacci, Chiara Barà, Andrea Zaccaro, Francesca Ferri, Riccardo Pernice, Luca Faes

**Affiliations:** ^1^ Department of Engineering, University of Palermo, Palermo, Italy; ^2^ Department of Neuroscience, Imaging and Clinical Sciences, “G. d’Annunzio” University of Chieti-Pescara, Chieti, Italy; ^3^ Institute for Advanced Biomedical Technologies (ITAB), “G. d’Annunzio” University of Chieti-Pescara, Chieti, Italy

**Keywords:** network physiology, information dynamics, information storage, time series analysis, autoregressive models, recursive least squares, electroencephalogram (EEG), heartbeat-evoked responses

## Abstract

Network Physiology is a rapidly growing field of study that aims to understand how physiological systems interact to maintain health. Within the information theory framework the information storage (IS) allows to measure the regularity and predictability of a dynamic process under stationarity assumption. However, this assumption does not allow to track over time the transient pathways occurring in the dynamical activity of a physiological system. To address this limitation, we propose a time-varying approach based on the recursive least squares algorithm (RLS) for estimating IS at each time instant, in non-stationary conditions. We tested this approach in simulated time-varying dynamics and in the analysis of electroencephalographic (EEG) signals recorded from healthy volunteers and timed with the heartbeat to investigate brain-heart interactions. In simulations, we show that the proposed approach allows to track both abrupt and slow changes in the information stored in a physiological system. These changes are reflected in its evolution and variability over time. The analysis of brain-heart interactions reveals marked differences across the cardiac cycle phases of the variability of the time-varying IS. On the other hand, the average IS values exhibit a weak modulation over parieto-occiptal areas of the scalp. Our study highlights the importance of developing more advanced methods for measuring IS that account for non-stationarity in physiological systems. The proposed time-varying approach based on RLS represents a useful tool for identifying spatio-temporal dynamics within the neurocardiac system and can contribute to the understanding of brain-heart interactions.

## 1 Introduction

The human body is composed of several physiological and organ systems, each one with its own distinct structural arrangement and complex functionality. This results in intricate and variable output dynamics that are characterized by complexity and fluctuations (Bashan et al., 2012; Schmal et al., 2022). The fundamental principles of physiology and clinical medicine often follow a reductionist approach that focuses on the structural organization and dynamics of individual organ systems when evaluating health and disease. On the other hand, the emerging field of Network Physiology (NP) combines empirical and theoretical knowledge from various disciplines to gain insights into the dynamic interactions of diverse organs, physiological systems, and sub-systems as a network. It encompasses extensive data analysis, modeling approaches, and clinical applications to comprehend how these interactions occur in different contexts. The goal is to understand how these interactions manifest at the cellular, organism, and systemic levels, giving rise to diverse physiological states and functions in both health and disease (Ivanov 2021). In recent years, there has been a growing body of evidence emphasizing the significance of examining the functional interactions between the brain and the heart. For instance, cardiac arrhythmias have been identified as a frequent cause of ischemic attacks (Pyner, 2014). Furthermore, cognitive disorders can arise from atrial fibrillation (Sabatini et al., 2000), even without evident stroke (Dorrance and Fink, 2011). Conversely, brain disorders such as stroke and epilepsy, believed to be triggered by environmental stressors, can give rise to cardiovascular disorders and have been demonstrated to induce both experimental and clinical cardiac arrhythmias (Wilkinson et al., 1998). To better elucidate these intricate mechanisms of neuroautonomic control several approaches have been developed which are mainly based on the study of the coupling between time series representative of the heart and the brain activities. The coupling strength can be captured, for instance, through the use of information theoretic measures or, alternatively, directly from the structure of the cross-correlation function. These approaches have been highly effective in comprehending and characterizing brain-heart dynamics in various experimental conditions, including wake and sleep (Bashan et al., 2012; Faes et al., 2014; Lin et al., 2016), physiological stress and rest (Antonacci et al., 2020; Pernice et al., 2021; Sciaraffa et al., 2021), mental workload and relaxed conditions (Widjaja et al., 2015), as well as emotion elicitation and neutral states (Schulz et al., 2013; Valenza et al., 2016; Greco et al., 2019). An alternative often-used approach is the study of heartbeat-evoked potentials (HEPs) (Schandry et al., 1986), which are responses in the cortex that are synchronized with contractions in the ventricles of the heart and can be identified by the R-peak in an electrocardiogram (ECG) (Al et al., 2020). Specifically, brain signals are segmented and timed with respect to the ECG R-peaks, and the relative potential is obtained through averaging (Luft and Bhattacharya, 2015; Coll et al., 2021). While it is believed that HEPs are connected to the neural processing of cardiac activity and can serve as an indicator of the heart-to-brain directional interaction, the interpretation of HEPs is still a matter of debate in the scientific community, as their physiological significance and neural mechanisms are not fully understood (Catrambone and Valenza, 2021). Recently, an approach to explore the brain responses evoked by the heartbeats from an information-theoretic perspective was introduced (Barà et al., 2023). This approach quantifies the regularity of the electroencephalographic (EEG) signals in different phases of the cardiac cycle through the computation of a local version of the Information Storage (IS), a measure which reveals the information content of the EEG signal at each time instant (Lizier et al., 2012). The IS can be defined as the information contained in the past history of a stochastic process that can be used to predict its future. Thus, it allows to measure the regularity and predictability of a dynamic process and, under Gaussian assumption, can be estimated within the identification of a simple linear model (Barnett et al., 2009; Faes et al., 2015). Moreover, this quantity is recognized as one of the three key component processes constituting every act of information processing in a network of interacting systems (i.e., information storage, transfer, and modification) (Wibral et al., 2014). Within this context, IS assumes a pivotal role in studying the dynamics of numerous processes (Faes et al., 2019) and has been used to study the internal dynamics of the human brain (Wibral et al., 2014), the cardiovascular (Faes et al., 2016) and the cardiorespiratory (Faes et al., 2015) systems.

Both approaches, the HEP and the local IS, aim to investigate brain-heart interactions focusing on the impact that the heartbeat has on the EEG dynamics. However, in spite of their potential, the local IS and evoked potentials may not be sufficient to take into account the transient characteristics of the EEG signals triggered by an external stimulus due to the stationarity assumption required for its calculation (Stramaglia et al., 2021; Barà et al., 2023). To address this limitation, a time-varying (TV) approach can be used to identify the transient pathways in the information stored in the brain as a response to the heart activity. This approach does not require stationary signals, and relies on an estimation algorithm which is a recursive version of the least squares analysis, called recursive least squares (RLS), involving a time-varying identification of a linear autoregressive (AR) model (Haykin, 2002). In the literature, the RLS algorithm has been used for connectivity estimation in both time and frequency domains to analyse the EEG signals. Specifically, Möller et al. (2001) proposed an application of RLS algorithm with forgetting factor for the coherence estimation to study the connectivity between pairs of EEG signals; Hesse et al. (2003) introduced the same algorithm for the time-varying estimation of the Granger causality to study the processing of information in the human brain during the execution of a color-word Stroop test; Astolfi et al. (2008) proposed the use of the same RLS algorithm for the estimation of directed transfer function and partial directed coherence with application to EEG signals for the study of event related potentials; Milde et al. (2010) compared the RLS algorithm with an approach based on Kalman filter to study the processing of evoked brain potentials from high dimensional data.

In this work, we exploit the RLS in combination with an iterative solution of the well-known Yule-Walker equations, to estimate time-specific IS in a non-stationary environment. In different simulation settings, the proposed approach is tested and its performances in the estimation of IS are evaluated. Alongside with this methodological advancement, from an applicative point of view, we then investigate the behaviour of the time-varying approach on EEG recordings to analyze the regularity of the neural activity timed with the heartbeat so as to assess brain-heart interactions.

The code necessary to compute the time-specific IS is collected in the Time-VaryingIS Matlab toolbox, described in the Supplementary Material and freely available for download at https://github.com/YuriAntonacci/Time-VaryingIS.

## 2 Materials and methods

This section outlines the methodological approaches utilized for assessing the information stored in a random process in a time-resolved way, under non-stationary conditions, based on estimating time-varying predictability measures using linear models identified via recursive least squares analysis.

### 2.1 Information-theoretic Preliminaries

Given a random variable *V*, the Shannon entropy is defined as H(V)=−E[log⁡p(v)] , where *p*(*v*) is the probability density function of *V* measured for the outcome *v*, and E[⋅] is the expectation operator computing the statistical average over all possible values *v* taken by *V*. A more specific quantity is the information content of the single outcome *v*, defined as *h*(*v*) = − log  *p*(*v*). This measure allows a *local* analysis of the information content of a random variable, i.e., an analysis focused on a specific outcome of the variable, while the Shannon entropy can be interpreted as a *global* measure, corresponding to the average information content across all outcomes, i.e., H(V)=E[h(v)] . These concepts can be generalized to any information-theoretic measure where a global measure can be interpreted as the statistical average of its local equivalent. For instance, the conditional entropy (CE) of variable *V* with respect to another variable *W* quantifies the residual information about *V* when *W* is known, representing the average level of uncertainty that persists about *V* when the outcomes of *W* are known: H(V|W)=E[h(v|w)] , where *h* (*v*|*w*) = − log  *p* (*v*|*w*) is the local CE. Likewise, the mutual information (MI) measures the information shared by the variables *V* and *W*, expressed as the average amount of uncertainty about one variable that can be resolved by the knowledge of the other: I(V;W)=E[i(v;w)] , where *i* (*v*; *w*) = log [*p* (*v*, *w*)/(*p*(*v*)*p*(*w*))] is the local MI. Note that entropy, CE and MI are linked to each other in both their local and global formulations, i.e., *h*(*v*) = *i* (*v*; *w*) + *h* (*v*|*w*) and *H*(*V*) = *I*(*V*; *W*) + *H*(*V*|*W*). Note also that, while the MI is always non-negative, the local MI can assume either positive or negative values; in the latter case, learning the outcome *w* for the variable *W* is interpreted as misinformative about the specific outcome *v* of the variable *V* (Lizier et al., 2012).

### 2.2 Information storage

Let us take into account a dynamic system Y , whose progression over time is governed by the discrete-time stochastic process *Y* = *Y*_*n*_ with n∈Z representing the temporal index. The scalar variable *Y*_*n*_ indicates the present state of the process, while the vector variable $Y_{n}^{-}=\left[Y_{n-1}Y_{n-2}\ldots \right]$ is taken to represent the past states of the process. The information storage (Lizier et al., 2012; Wibral et al., 2014) has been defined as the information in an agent, process or variable’s past that can be used to predict its future (Lizier et al., 2012). Such storage refers to how dynamical systems store, structure and transform historical and spatial information and can be interpreted as a measure of the process regularity defined as:

$$S_{Y,n}=I\left(Y_{n};Y_{n}^{-}\right)=E\left[\log\frac{p\left(y_{n},y_{n}^{-}\right)}{p\left(y_{n}\right)p\left(y_{n}^{-}\right)}\right],$$ (1)

where *y*_*n*_ and $y_{n}^{-}$ refer to realizations of *Y*_*n*_ and $Y_{n}^{-}$ , respectively. The IS measures the predictability of the process at time *n* by quantifying the average level of uncertainty about the current state of the process, *Y*_*n*_, that can be resolved by the knowledge of its past states, $Y_{n}^{-}$ .

Assuming that *Y* is a Markov process with finite memory of order *p*, its whole past history can be truncated using *p* time steps, i.e., using the *p* − dimensional variable $W_{n}\in R^{p\times 1}$ such that $Y_{n}^{-}\approx W_{n}={\left[Y_{n-1},\ldots ,Y_{n-p}\right]}^{⊤}$ . Then, the current state *Y*_*n*_ can be predicted as a linear combination of the past states by means of a linear time-varying AR (TV-AR) model:

$$Y_{n}=\overset{p}{\underset{k=1}{\sum }}a_{k,n}Y_{n-k}+U_{n},$$ (2)

where *U*_*n*_ is the prediction error and *a*_*k*,*n*_ is the AR coefficient at the time instant *n* that relates the present state of the process with its past state at lag *k*. Then, under Gaussian assumption of *Y*_*n*_, its entropy can be expressed as (Cover, 1999): $H\left(Y_{n}\right)=\frac{1}{2}\log\left(2\pi e\cdot \sigma_{Y_{n}}^{2}\right)$ , where $\sigma_{Y_{n}}^{2}$ represents the variance of the process at the time instant *n*. Moreover, if *Y*_*n*_ and *W*_*n*_ are jointly Gaussian the conditional entropy of *Y*_*n*_ given *W*_*n*_ can be expressed as (Barnett et al., 2009): $H\left(Y_{n}|W_{n}\right)=\frac{1}{2}\log\left(2\pi e\cdot \sigma_{U_{n}}^{2}\right)$ , where $\sigma_{U_{n}}^{2}$ is the variance of the prediction error *U*_*n*_ at the relevant time instant. Thus, the relation stated in Eq. 1 can be re-written as:

$$S_{Y,n}=H\left(Y_{n}\right)-H\left(Y_{n}|W_{n}\right)=\frac{1}{2}\log\frac{\sigma_{Y_{n}}^{2}}{\sigma_{U_{n}}^{2}},$$ (3)

providing a viable way to compute the information stored in the process at each specific time point. In particular, under ergodicity and stationarity assumptions, the information stored in the process *Y* is the same at each time *n* (*S*_*Y*,*n*_ = *S*_*Y*_) returning the well-known time-invariant IS measure (Lizier et al., 2011). On the other hand, whether the process is non-stationary, the time-varying version of the IS defined in (3) can be computed as described in the following section. Note that, the definitions of MI and CE given in Section 2.1 are used in this work to characterize the interaction between different random variables taken from the same individual random process.

### 2.3 Linear parametric estimation under non-stationarity assumption: time specific information storage

Given the analyzed stochastic process *Y*, the TV-AR model reported in Eq. 2 can be formulated in compact form as: $Y_{n}=A_{n}^{⊤}W_{n}+U_{n}$ , where $A_{n}={\left[a_{1,n},\ldots ,a_{p,n}\right]}^{⊤}\in R^{p\times 1}$ is the vector of the TV-AR coefficients to be estimated. For this estimation task we use a recursive implementation of the least squares method (RLS) which involves the minimization of the cost function $J\left(A_{n}\right)=\sum_{i=1}^{n}{\left(1-c\right)}^{n-i}\|Z_{i}{\|}^{2}$ , where $A_{n}\in R^{p\times n}$ is a matrix containing the temporal evolution of the *p* coefficients and $Z_{i}=Y_{i}-W_{i}^{⊤}A_{i-1}$ is a scalar value denoting the *a-priori* estimation error computed as difference between the desired response *Y*_*i*_ and the estimated response ${\hat{Y}}_{i}$ . The term (1 − *c*)^*n*−*i*^ is the exponential weighting factor, or forgetting factor, with 0 ≤ *c* < 1 which ensures that the data in the distant past are “forgotten” in order to afford the possibility of following the statistical variations in non-stationary conditions; note that when *c* = 0 we have the ordinary least squares prediction (Möller et al., 2001). In particular, this parameter controls the trade-off between the adaptation speed and the variance of the estimate.

### 2.3.1 Time-varying autoregressive model identification

The RLS algorithm to estimate the vector of AR coefficients consists in the following computation steps (Milde et al., 2010): 1) choose a value for the adaptation factor *c* and an order of the TV-AR model *p*; 2) define $A_{p}={\left[a_{1,p},\ldots ,a_{p,p}\right]}^{⊤}=0\in R^{p\times 1}$ as the vector of coefficients at time *p* and $\Phi_{p}^{w}=0\in R^{p\times p}$ as the correlation matrix of the lagged term stored in *W*_*p*_; (iii) for *n* = *p* + 1 to *N* repeat the following steps.

$$\Phi_{n}^{w}=\left(1-c\right)\Phi_{n-1}^{w}+W_{n}W_{n}^{⊤}$$ (4a)

$$K_{n}={\left(\Phi_{n}^{w}\right)}^{-1}W_{n}$$ (4b)

$$Z_{n}=Y_{n}-W_{n}^{⊤}A_{n-1}$$ (4c)

$$A_{n}=A_{n-1}+K_{n}Z_{n},$$ (4d)

where $K_{n}\in R^{p\times 1}$ is the so-called gain vector and $Z_{n}\in R^{1\times 1}$ is intended as the *a-priori* estimation error that can be viewed as a “tentative” value of error before updating the AR coefficients vector. For a detailed mathematical derivation of the RLS solution as described in (Haykin, 2002), we refer the interested reader to the Supplementary Material.

To complete the identification procedure of the TV-AR model (2) it is necessary to obtain a recursion for the innovation variance. In particular, when 0 < *c* < 1, following the results obtained in (Grieszbach et al., 1994), it is possible to obtain a recursive estimation of the time-varying innovation variance $\sigma_{U_{n}}^{2}$ as follows:

$$\sigma_{U_{n}}^{2}=\sigma_{U_{n-1}}^{2}+c\left(Z_{n}^{2}-\sigma_{U_{n-1}}^{2}\right).$$ (5)

To complete the estimation procedure of the time specific IS, as defined in Eq. 3, we need also a recursive estimation of the process variance $\sigma_{Y_{n}}^{2}$ , which is derived by assuming Gaussianity of the process *Y* and as initial condition $\sigma_{U_{0}}^{2}=0$ , by the procedure reported in the following. The autocovariance of the process (2) is related to the TV-AR parameters via a time varying version of the Yule-Walker equations (Lütkepohl, 2013):

$$\Gamma_{k,n}=\overset{p}{\underset{l=1}{\sum }}a_{l,n}\Gamma_{k-l,n}+\delta_{k0}\sigma_{U_{n}}^{2},$$ (6)

where $\Gamma_{k,n}=E\left[Y_{n}Y_{n-k}\right]$ represents the autocovariance of the process defined at each lag *k* ≥ 0, and *δ*_*k*0_ is the delta of Kronecher. In order to determine the autocovariance of the process for each lag *k* and for each time instant *n*, the TV-AR model can be written compactly (Faes et al., 2015) as $S_{n}^{p}=A_{n}^{p}S_{n-1}^{p}+U_{n}^{p}$ where:

$$S_{n}^{p}={\left[Y_{n}\;Y_{n-1}\;\cdots \;Y_{n-p+1}\right]}^{⊤},A_{n}^{p}=\left[\begin{array}{cccc} a_{1,n} & \cdots & a_{p-1,n} & a_{p,n}\\ 1 & \cdots & 0 & 0\\ \vdots & \ddots & \vdots & \vdots \\ 0 & \ldots & 1 & 0 \end{array}\right],U_{n}^{p}=\left[\begin{array}{c} U_{n}\\ 0\\ \vdots \\ 0 \end{array}\right]$$ (7)

Specifically, the covariance matrix of $S_{n}^{p}$ can be expressed as a discrete-time Lyapunov equation:

$$\Gamma_{0,n}^{p}=E\left[S_{n}^{p}{S_{n}^{p}}^{{\;}^{⊤}}\right]=A_{n}^{p}\Gamma_{0,n}^{p}{A_{n}^{p}}^{{\;}^{⊤}}+\Lambda_{n}^{p},$$ (8)

where $\Lambda_{n}^{p}=E\left[U_{n}^{p}{U_{n}^{p}}^{{\;}^{⊤}}\right]\in R^{p\times p}$ is the covariance matrix of $U_{n}^{p}$ . Solving (8) for $\Gamma_{0,n}^{p}$ allows to obtain autocovariance values for lag between 0 and *p* − 1, Γ_0,*n*_ … Γ_*p*−1,*n*_. Knowing that $\Gamma_{0,n}=\sigma_{Y_{n}}^{2}$ , the innovation variance can be directly extracted from the autocovariance structure of the process for each considered time instant *n*.

To summarize, to compute the time specific IS we proceed as follows: 1) starting from the TV-AR parameters $\left(a_{1,n},\ldots ,a_{p,n},\sigma_{U_{n}}^{2}\right)$ estimated via the RLS solution, compute the autocovariance of the process $\Gamma_{0,n}^{p}$ solving the Lyapunov Eq. 8; 2) pick the element (1,1) of the computed autocovariance to obtain a time specific estimation of the variance of the process $\sigma_{Y_{n}}^{2}$ ; (iii) compute the time specific IS following Eq. 3.

## 3 Simulation study

This section explores the behavior of the time-varying IS, by modifying the statistical structure of a first-order autoregressive process (AR (1)) over time changing the value of the autoregressive coefficient according to predefined waveforms. We design a univariate TV-AR process defined by the following equation: *Y*_*n*_ = *a*_1,*n*_ *Y*_*n*−1_ + *U*_*n*_, where *U*_*n*_ is a zero-mean white Gaussian noise with variance $\sigma_{U_{n}}^{2}$ , with *a*_1,*n*_ representing the coupling strength between the past state and the present state of *Y*. Assuming a sampling frequency *f*_*s*_ = 500 Hz, one realization of the AR (1) process was generated for a duration of 10*s*, resulting in a total of *N* = 5000 data samples. The coefficient *a*_1,*n*_ has been set to vary over time as 1) a periodic square waveform and 2) a periodic sinusoidal waveform, both oscillating in amplitude between 0.3 and 0.9, with frequency of the oscillation *f* = 0.3 Hz. The duty cycle was set to 50% for the square waveform. We performed two different analyses: in the first, one single realization of the TV-AR process was generated for each of the two periodic waveforms, carrying out the IS estimation with different values of forgetting factor (1 − *c* ∈ {0.95, 0.97, 0.99})(Möller et al., 2001); in the second, we considered only the periodic square waveform, to evaluate with more detail the effects of the forgetting factor on the estimation of the time-varying IS. The estimates of IS were performed when the forgetting factor varied between 0.9 and 0.999 with a step size of 0.001. Then, two conditions were considered, named ON when the amplitude of the square waveform was 0.9, and OFF when the amplitude was 0.3, leading to theoretical values of IS equal to 0.83 (ON, $S_{y-th}^{+}$ ) and 0.05 (OFF, $S_{y-th}^{-}$ ), respectively. For each forgetting factor value and for each condition, different performance parameters were computed: 1) the bias defined as the difference between the average value of the estimated IS and the true theoretical value, BIAS $=E\left[{\hat{S}}_{y,n}\right]-S_{y-th}$ (BIAS-ON, BIAS-OFF); 2) the estimation variance VAR $=E\left[{\left({\hat{S}}_{y,n}-E\left[{\hat{S}}_{y,n}\right]\right)}^{2}\right]$ (VAR-ON, VAR-OFF); 3) the average values of bias and variance (BIAS, VAR) between the two conditions computed as the average of absolute values for the bias and as a simple average for the variance; 4) the rise time computed as the time required for the estimate of IS to rise from the 10% of the lowest value, $S_{y-th}^{-}$ , to the 90% of the highest value $S_{y-th}^{+}$ ; 5) the fall time computed as the time required for the estimate of IS to fall from the 90% of the highest value, $S_{y-th}^{+}$ , to the 10% of the lowest value $S_{y-th}^{-}$ ; 6) the average value between the rise and fall time, named as Rise-Fall time (RFT).

**FIGURE 1:**
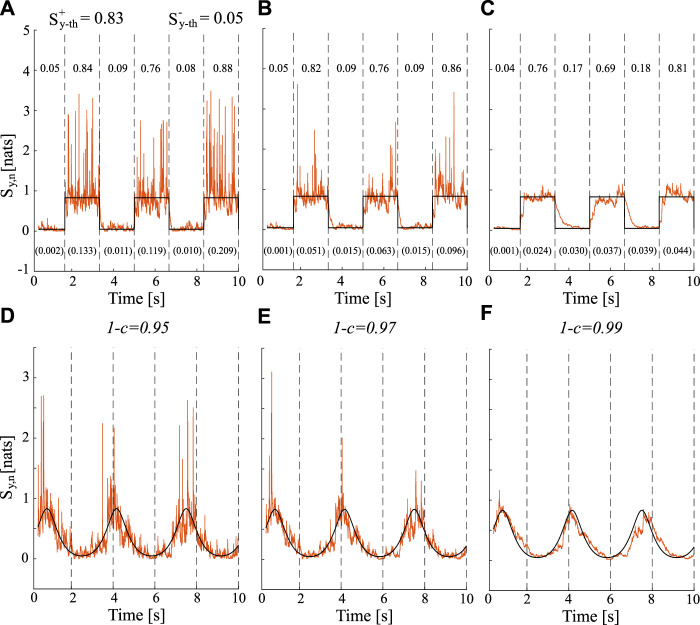
Estimated and theoretical trends (orange lines and black lines respectively) for time-varying IS computed for different values of forgetting factor (0.95, panels **(A and D)**; 0.97, panels **(B and E)**; 0.99, panels **(C and F)** when *a*_1,*n*_ varies over time as: a square waveform (top row) and a sinusoidal waveform (bottom row). For the square waveform, the theoretical true values of the IS are $S_{y-th}^{+}$ and $S_{y-th}^{-}$ for the highest and lowest steady states, and the average values and variance of the IS estimates are reported on the top and on the bottom (in brackets) of the figure, respectively.

Figure 1 displays the theoretical (black lines) and estimated (orange lines) trends of the time-varying IS obtained for different values of forgetting factor 1 − *c* ∈ {0.95, 0.97, 0.99} when *a*_1,*n*_ oscillates as a square (Figures 1A–C) or a sinusoidal (Figures 1D–F) waveform. The coupling strength (*a*_1,*n*_) between the past state, *Y*_*n*−1_, and the present state, *Y*_*n*_, controls the amount of information contained in the system *Y*. In particular, the highest and the lowest values of *a*_1,*n*_, i.e., 0.9 and 0.3, indicate the greatest or lowest amount of information stored in the system, respectively.

The results show how the estimates of TV-IS follow the true theoretical values exhibiting different behaviors in terms of bias and variance depending on the value of the forgetting factor.The trends depicted in Figure 1 demonstrate that the increase of the forgetting factor from 0.95 (Figures 1A, D) to 0.99 (Figures 1C, F) is associated with a reduction of the estimation variance regardless of the waveform considered. This can be clearly observed by looking at the variance of the estimate reported in brackets at the bottom of the Figures 1A–C when the square waveform is considered. Moreover, the increment of the adaptation factor *c* (which corresponds to a decrement of the forgetting factor) affects the adaptation speed to transitions which increases as well. Moreover, the average values of the IS estimated within each steady state of the square waveform were consistently close to the theoretical values ( $S_{y-th}^{+}$ , $S_{y-th}^{-}$ ) even if a slight underestimation occurs when increasing the forgetting factor.

**FIGURE 2:**
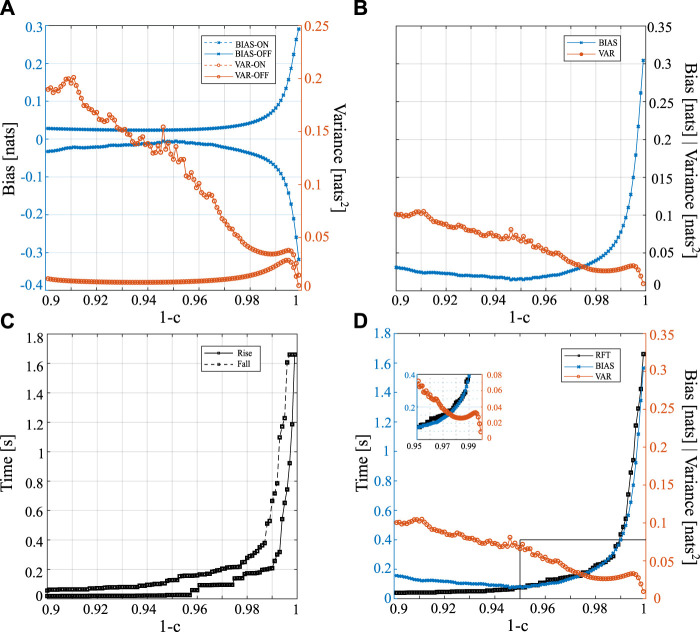
Trends of the various performance parameters of the procedure for the estimation of the time-varying IS obtained for different values of forgetting factor 1 − c in the interval [0.9, 0.999] (step size = 0.001). The time-varying IS was simulated by varying over time *a*_1,*n*_ as a square periodic waveform. Analysis of bias and variance in the ON and OFF conditions taken separately **(A)** or jointly and computed as average trends **(B)**. **(C)** Analysis of the rise and fall times used as a measure of adaptation speed to a transition. **(D)** Average trends for bias (BIAS), variance (VAR) and rise-fall time (RFT).

Figure 2 presents the trends of bias, variance, rise time, and fall time for different values of the forgetting factor in the range of 0.9–0.999 (1 − *c* = 1 was excluded since it represents the OLS solution in a stationary condition). The trends of bias and variance obtained for the two steady states (ON-OFF) are reported separately and then averaged in Figures 2A, B, respectively. The analysis of the bias reveals that, as the forgetting factor increases, an increasing overestimation in the OFF condition occurs, whereas in the ON condition there is an increasing underestimation. These trends are particularly noticeable when the forgetting factor exceeds 0.95, as the curve takes on an exponential shape above this value. The variance in the OFF condition appears to be negligible when the forgetting factor is set to 0.97. As the value of 1 − *c* increases in the range of 0.99–0.996, the variance reaches a peak and subsequently decreases. On the other hand, the ON condition exhibits a marked descending trend in variance, which resembles the OFF phase trend when 1 − *c* exceeds 0.99. Figure 2B illustrates the evolution of the overall variance and bias as a function of 1 − *c*, revealing opposite trends: the variance decreases as 1 − *c* increases, while the bias increases. Figure 2C shows the rise and fall times required for the response to transition, which increase when augmenting 1 − *c*. Specifically, the rise and fall times approximately increase from 200 ms for 1 − *c* = 0.98–1.6 s for 1 − *c* = 0.996. The results presented in Figures 2A–C are summarized in Figure 2D, which displays the average trends of bias (BIAS), variance (VAR), and rise-fall time (RFT) as a function of the forgetting factor. The trends of RFT and BIAS exhibit almost identical shapes, both showing a marked increase starting from 1 − *c* = 0.95, whereas the estimation variance shows the opposite trend. It is worth noting that the three curves intersect at a specific forgetting factor value between 0.97 and 0.98, as illustrated in the inset of Figure 2D.

## 4 Analysis of brain-heart interplay

This section employs the time-varying IS measure defined in Section 2 to investigate the influence of the heartbeat on cortical dynamics measured from scalp EEG. The regularity of neural activity in relation to the heartbeat is analyzed by calculating the mean and variability of the time-varying IS within specific temporal windows that coincide with each identified heartbeat from the ECG. The objective is to monitor changes in the predictability of EEG signals over time and explore the connection between cardiac activity and cortical processing of the heartbeat.

### 4.1 Data acquisition and pre-processing

**FIGURE 3:**
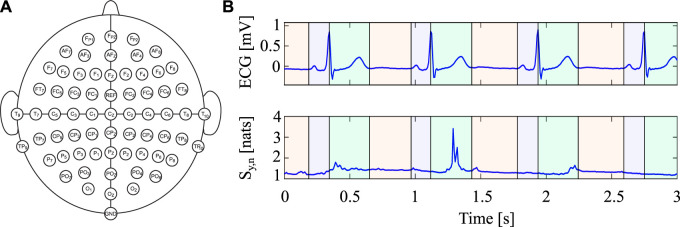
**(A)** Overview of the EEG electrode montage according with the international standard 10/20 highlighting the position of the 62 EEG electrodes covering the scalp of the subjects. **(B)** Portion of the ECG signal (top row) and corresponding time-varying (bottom row) Information Storage within the three intervals obtained after the segmentation of the cardiac cycle (*I*_1_ in green, *I*_2_ in red and *I*_3_ in purple).

In this investigation, 20 healthy individuals aged between 25 and 50 years (14 females; age: 25.21 ± 2.64 years), who were not undergoing psychopharmacological therapy or taking any extended medication, were monitored simultaneously from EEG and ECG signals. The acquisitions have been performed in the early afternoon between 2.00 p.m. and 4.00 p.m. to guarantee consistent and replicable experimental settings for all participants. The BrainAmp amplifier (BrainCap MR, Brain Vision, LLC) was utilized to obtain 64 EEG channels according to the international extended 10/20 system, with the *FC*_*Z*_ electrode serving as reference and the Inion electrode (*I*_*z*_) as ground (as shown in Figure 3A) (Zaccaro et al., 2022). The ECG signal was recorded using a one-lead system (BIOPAC System, INC), with both signals having a sampling frequency of 2 kHz. During the experimental protocol, participants were requested to rest with their eyes open, staring at a fixation cross at the center of a computer screen, and permitting their thoughts to wander for around 10 min. The study was endorsed by the Institutional Review Board of Psychology, Department of Psychological, Health and Territorial Sciences, “G. d’Annunzio” University of Chieti-Pescara (Protocol Number 44_26_07_2021_21016), and adhered to the Italian Association of Psychology and the Declaration of Helsinki guidelines, as well as its subsequent amendments. Each subject provided written informed consent. For additional information about the acquisition system and dataset, see Zaccaro et al. (2022).

The EEG signals were processed offline using the EEGLAB signal processing Toolbox of MATLAB (Delorme and Makeig, 2004). A Hamming window FIR filter was applied to the signals using a bandpass filter with cutoff frequencies ranging from 0.5 to 40 Hz. The signals were manually cleaned to eliminate artifacts and noise that resulted from movements or improper electrode-skin contact. Any noisy channel was spherically interpolated. To decrease the influence of artifacts on the EEG signals, Independent Component Analysis (ICA) was conducted using the fastICA algorithm (Hyvärinen and Oja, 2000). Subsequently, the signals were subsampled to 128 Hz to reduce redundancy between adjacent samples before undergoing information-theoretic analysis, an re-referenced to the average of all channels (Zaccaro et al., 2022). In the case of the ECG signals, the R-peaks were detected using a modified version of the Pan-Tompkins algorithm (Pan and Tompkins, 1985), and a specifically-designed threshold-based peak detection algorithm was employed to extract T and P waves. Two subjects were not included in further analyses due to the presence of artifacts. The final length of the recordings was 423.47 ± 27.5 s (range 320.26 s − 429.29 s).

### 4.2 Data analysis

The approach outlined in Section 2 was used to calculate the time-varying IS for all EEG signals obtained from each subject. To perform the computation of time-varying IS using the RLS approach, following our previous work on the same dataset (Barà et al., 2023), the order *p* of the TV-AR model was set to 5. Different values of the forgetting factor (1 − *c* ∈ {0.95, 0.97, 0.99}) were used to investigate its effects on real data analysis.

For each EEG signal, the time-varying IS was analyzed separately in three different intervals defined by segmenting each cardiac cycle, as depicted in Figure 3B, and in accordance with Barà et al. (2023). To account for the impact of Cardiac Field Artifact (CFA) on EEG signals, three intervals were defined, as the electrical activity of the heart can affect the amplitude of EEG signals depending on the phase of the cardiac cycle as well as on the distribution of electrodes on the scalp (Dirlich et al., 1997). The first segment (*I*_1_) started at the R-peak of the ECG signal and ended 80 ms after the T-wave peak, the second segment (*I*_2_) started at the end of the first segment and ended 40 ms before the P-wave peak of the following cardiac cycle, while the third segment (*I*_3_) corresponded to the remainder of the cardiac cycle until the R-peak of the subsequent cycle. The second segment *I*_2_ was considered a low-CFA segment, while the impact of the artifact was more significant during the QRS complex and T-wave, especially for *I*_1_ and *I*_3_ (Dirlich et al., 1997). The analysis was performed on the first 300 heartbeats following short-term heart rate variability analysis guidelines (Shaffer and Ginsberg, 2017). Mean and standard deviation (STD) values of the time-varying IS were calculated for each electrode and subject within each of the three segments. Additionally, to provide a reference independent of time segmentation, mean and STD were also computed across the entire cardiac cycle (Global interval (G)).

### 4.3 Statistical analysis

For each EEG channel and for each subject, TV-IS was firstly averaged within the considered time interval (G, *I*_1_, *I*_2_, and *I*_3_) by obtaining 300 values (one for each cardiac cycle) that were then averaged to obtain a distribution across the 18 subjects. To obtain a distribution also for the standard deviation of the TV-IS, this procedure was repeated averaging the 300 values of STD obtained for each subject. These procedures resulted in a distribution for each parameter (MEAN and STD) and for each interval (G, *I*_1_, *I*_2_, and *I*_3_) across the 18 subjects. The described analysis was repeated changing the forgetting factor as in the previously described simulation study (1 − *c* ∈ {0.95, 0.97, 0.99}).

The statistical analysis aimed to compare the distributions of MEAN and STD of TV-IS measured across 18 subjects during the whole cardiac cycle and during each of the three intervals, testing the significance of the comparisons: G vs. *I*_1_, G vs. *I*_2_, and G vs. *I*_3_. A second analysis aimed to compare the distributions of MEAN and STD of the TV-IS measured across 18 subjects in the three intervals, testing the significance of the comparisons: *I*_1_ vs. *I*_2_, *I*_1_ vs. *I*_3_, and *I*_2_ vs. *I*_3_. For both analyses, paired Student’s t-test was used and Bonferroni correction for multiple comparisons was applied (*n* = 62 comparisons).

We computed also a measure of the effect size to assess the magnitude of the differences observed among the analyzed intervals for MEAN and STD. Denoting as $\mu_{Y_{1}}$ and $\mu_{Y_{2}}$ and with $\sigma_{Y_{1}}^{2}$ and $\sigma_{Y_{2}}^{2}$ the mean and the variance of the two equally sized distributions *Y*_1_ and *Y*_2_ obtained measuring the mean and the standard deviation of the TV-IS across subjects, the Cohen’s *d* measure was computed as (Sullivan and Feinn, 2012):

$$d=\frac{\mu_{Y_{1}}-\mu_{Y_{2}}}{\sqrt{\left(\sigma_{Y_{1}}^{2}+\sigma_{Y_{2}}^{2}\right)/2}}.$$ (9)

Typically a small effect size occurs for *d* = 0.2, whereas it is considered large when *d* = 0.8 (Sullivan and Feinn, 2012). To obtain a summary of the effect size, the values of Cohen’s *d* for all the EEG electrodes for which the test was statistically significant were averaged.

### 4.4 Results of real data analysis

**FIGURE 4:**
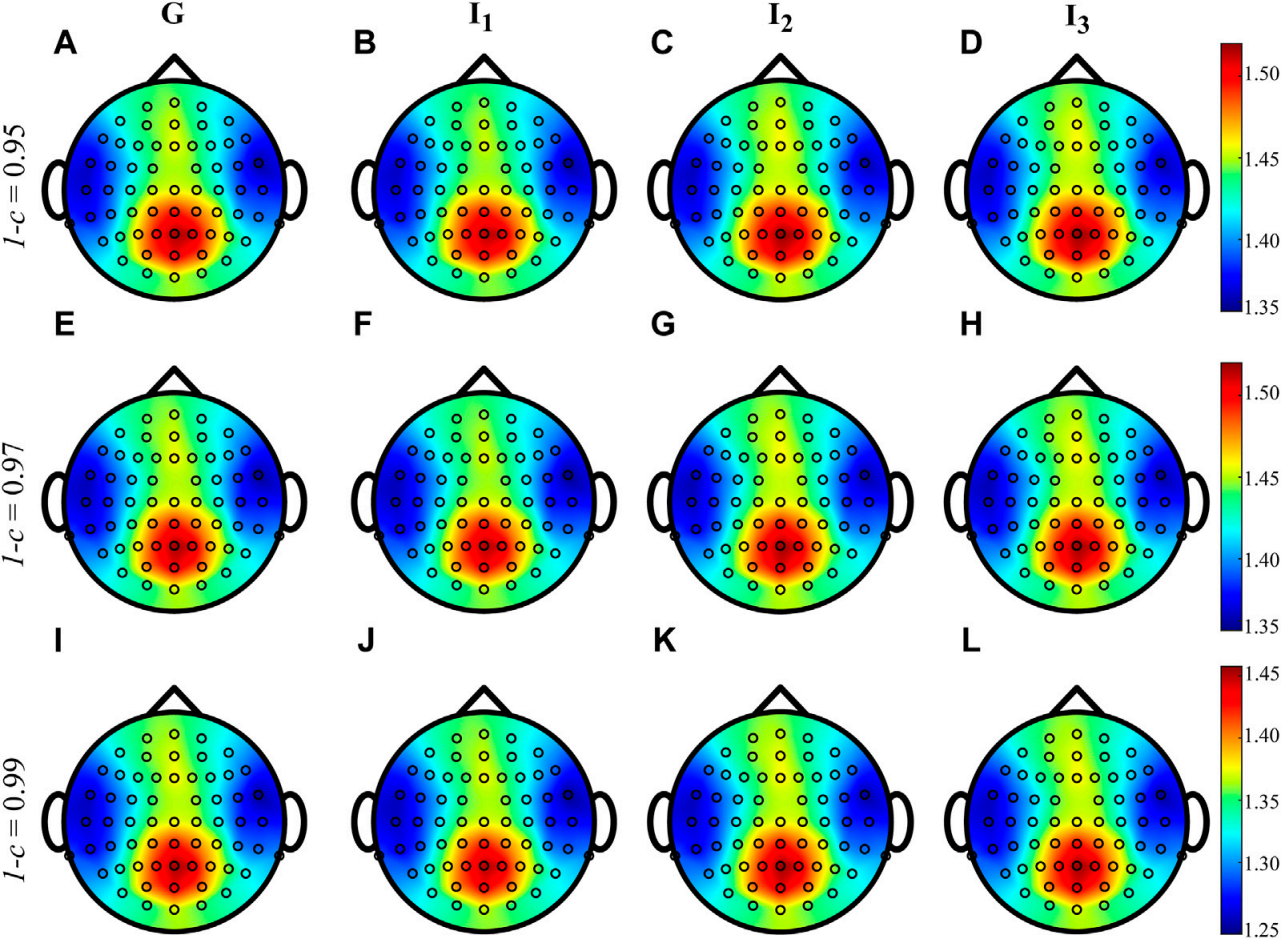
Grand average distributions over the scalp of the MEAN index, for each time interval, obtained by estimating time-varying IS of the EEG signals with different values of forgetting factor (0.95-**(A–D)**, 0.97-**(E–H)**, 0.99-**(I–L)**). G indicates the computation over the whole cardiac cycle whereas *I*_1_, *I*_2_ and *I*_3_ represent each analyzed interval taken as fraction of the cardiac cycle.

Figure 4 shows the grand average distributions over the scalp of the MEAN index computed over the whole cardiac cycle (listed as G, panels A, E, I) and within each interval *I*_1_, *I*_2_ and *I*_3_ for three different values of forgetting factor. The average trends shown by the time-varying approach when computed during G, *I*_1_, *I*_2_ and *I*_3_ are very similar to each other, irrespective of the value of the forgetting factor used for the estimation procedure. The values of IS vary between 1.25 and 1.45 nats when 1 − *c* = 0.99 and between 1.35 and 1.50 nats when 1 − *c* ∈ {0.95, 0.97}, with the information content which is mainly localized in the parietal and occipital areas with the involvement also of the frontal area of the scalp.

**FIGURE 5:**
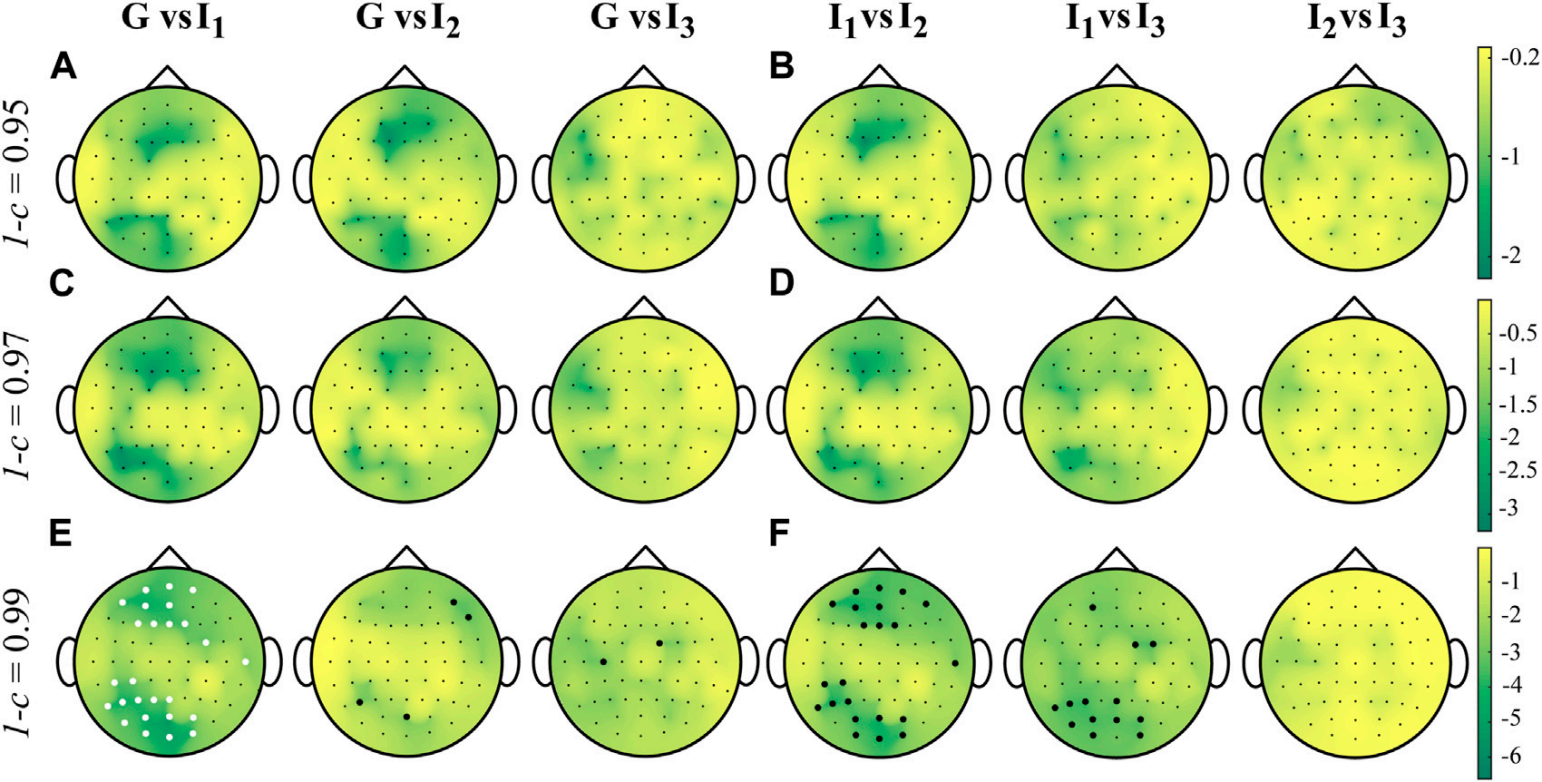
Distributions over the scalp of the logarithmic *p*-values obtained as a result of the statistical analysis carried out by comparing the distributions across the 18 subjects of the MEAN time-varying IS computed with values of forgetting 1 − *c* ∈ {0.95, 0.97, 0.99}, in all the time windows analyzed. Given two intervals *i* and *j*, white and black filled circles on a specific position over the scalp denote that the MEAN IS is significantly higher or lower during *i* and during *j*, respectively.

Figure 5 shows the distributions over the scalp of the logarithmic *p*-values obtained as a result of the statistical analyses described in Section 4.3, for the MEAN IS index computed for each subject, electrode and interval and for different values of the forgetting factor. The time-varying approach with forgetting factor 1 − *c* ∈ {0.95, 0.97} underlines no statistical significance between pairs of intervals. On the other hand, the use of a forgetting factor equal to 0.99 points out a diverse situation, with a large number of statistically significant differences in the MEAN IS between pairs of intervals. These differences are such that the MEAN IS is higher during G than *I*_1_ and lower during G than both *I*_2_ and *I*_3_ (panel E), and lower during *I*_1_ than *I*_2_ and *I*_3_, and during *I*_2_ than *I*_3_ (panel F). However, these differences, though statistically significant, are very little, as documented by the very low effect size that they produce. Indeed, the Cohen’s *d* assumes an average value over the scalp that is never greater than 0.05. When the forgetting factor is less than 0.99, the Cohen’s *d* is always below 0.03, irrespective of the pairs of intervals considered for the statistical analysis.

**FIGURE 6:**
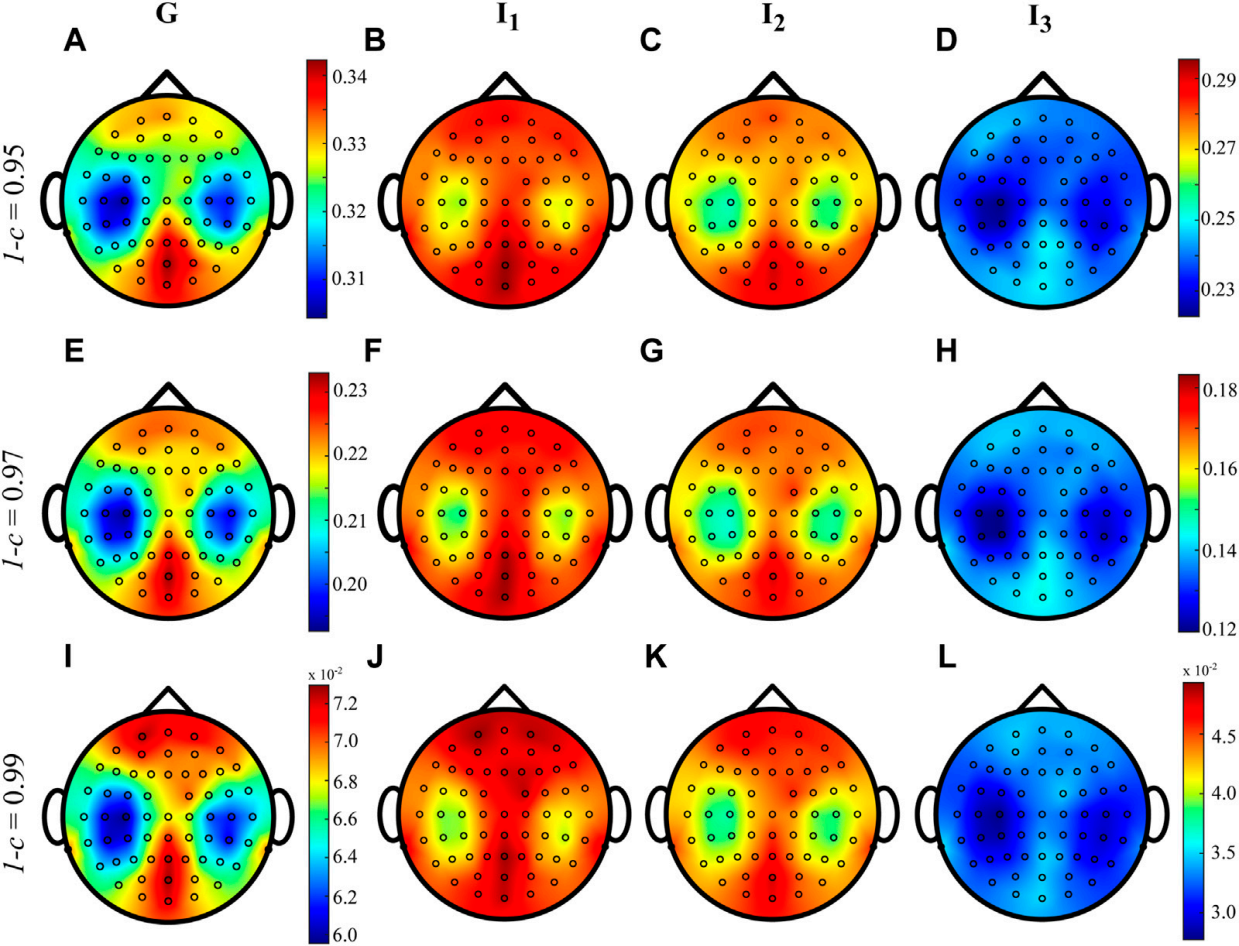
Grand average distributions over the scalp of the STD index, for each time interval, obtained by estimating time-varying IS of the EEG signals with different values of forgetting factor (0.95-**(A–D)**, 0.97-**(E–H)**, 0.99-**(I–L)**). G indicates the computation over the whole cardiac cycle whereas *I*_1_, *I*_2_ and *I*_3_ represent each analyzed interval taken as fraction of the cardiac cycle.

**FIGURE 7:**
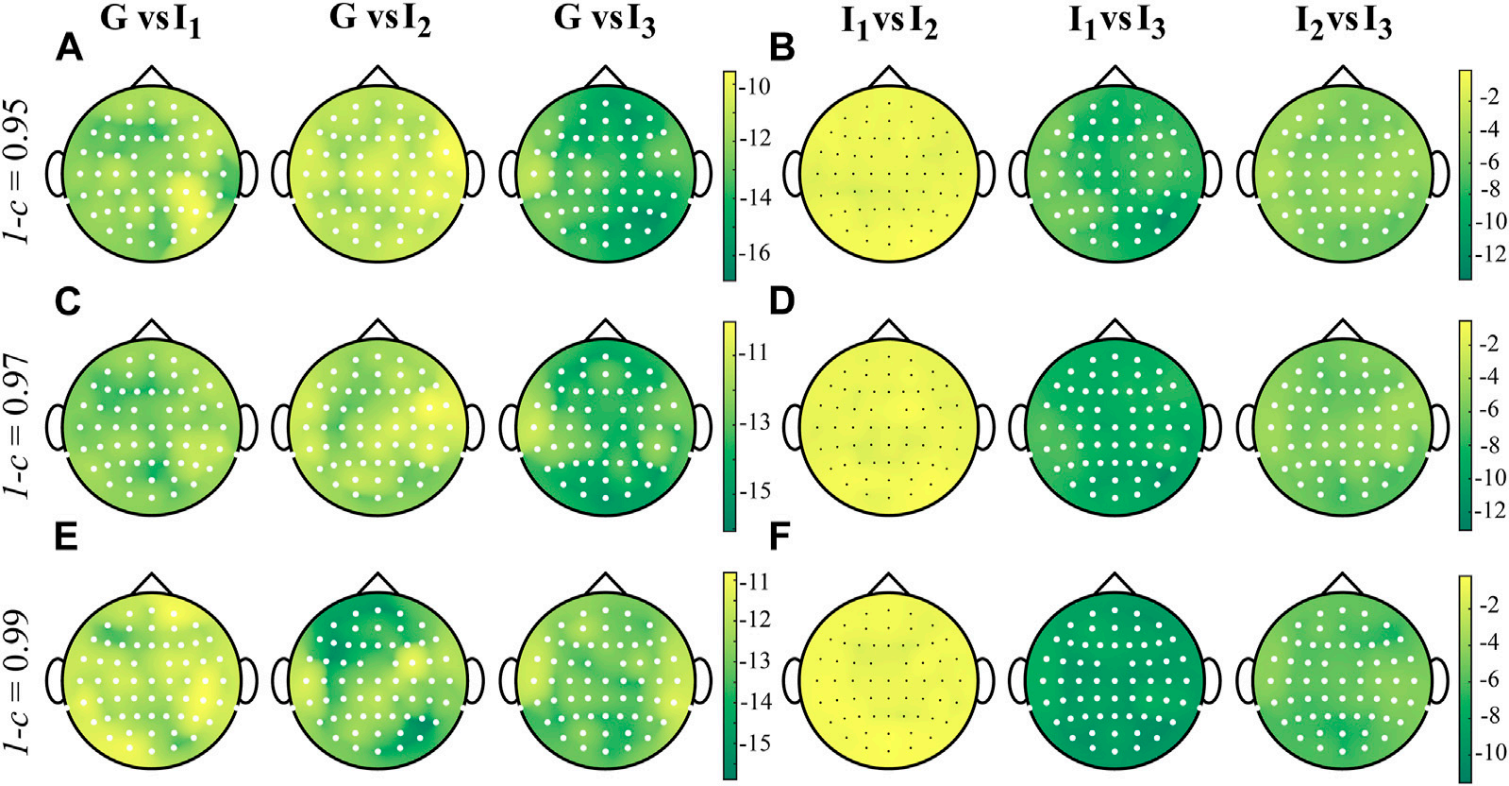
Distributions over the scalp of the logarithmic *p*-values obtained as a result of the statistical analysis carried out by comparing the distributions across the 18 subjects of the STD of the time-varying IS computed with values of forgetting 1 − *c* ∈ {0.95, 0.97, 0.99}, in all the time windows analyzed. Given two intervals *i* and *j*, white and black filled circles on a specific position over the scalp denote that the STD of the IS is significantly higher and lower during *i* and during *j*, respectively.

Figure 6 shows the grand average distributions over the scalp of the STD index computed over the whole cardiac cycle (G, panels A, E, I) and within each interval *I*_1_, *I*_2_ and *I*_3_ for three different values of forgetting factor. The values of STD increase while decreasing the forgetting factor, highlighting a higher variability of the IS associated with low forgetting factor. For all forgetting factors, the highest fluctuations of the IS are condensed in the frontal and parieto-occipital regions, while lower values of STD are observed in the central-temporal regions. These regional differences are observed for all intervals.The highest values of STD occur for the three analyzed cases in the global interval (panels A, E, I), while the standard deviation values become weaker moving from *I*_1_ to *I*_3_ (panels C–D, G-H, K-L). Figure 7 shows the distributions over the scalp of the logarithmic *p*-values obtained as a result of the statistical analysis carried out as described in Section 4.3 for the STD values extracted from each subject, electrode and interval. The standard deviation of the time-varying IS assumes a significantly higher value over the whole scalp during G than in each specific interval (panels A, C, E). When looking at the comparison between intervals, the STD does not vary significantly between *I*_1_ and *I*_2_ (except for only one electrode), while it decreases significantly moving from *I*_1_ to *I*_3_ and from *I*_2_ to *I*_3_ (panels B, D, F). The effect size measure confirms the relevance of the changes between intervals, as it is always higher than 1.5, independently from the value of the forgetting factor used for the estimation procedure (except while comparing *I*_1_ vs. *I*_2_ when *d* < 1 was obtained).

## 5 Discussion

This study aimed to introduce a novel approach to investigate the temporal evolution of the information stored in a physiological system. This approach exploits the RLS algorithm to estimate time-specific IS in a non-stationary environment. Its performances in the estimation of IS were explored in different simulation settings and then tested on neural signals related to different phases of the cardiac cycle to investigate brain-heart interactions.

### 5.1 Simulation study

The simulation study was conducted to analyze the effectiveness of the proposed approach to estimate the time-specific IS in controlled non-stationary conditions. To achieve this objective, we varied the TV-AR coefficient *a*_1,*n*_ over time using predefined square and sinusoidal waveforms in two simulated scenarios. The estimation procedure was repeated for three distinct values of forgetting factor (1 − *c* ∈ {0.95, 0.97, 0.99}) and the resulting trends were then compared with the true theoretical values. Then, we evaluated the impact of the forgetting factor on the estimation of IS by computing the bias, the variance, the fall time, and the rise time for different values of 1 − *c* in the interval [0.9, 0.999].

The estimation of IS using the RLS algorithm highlights its ability to accurately track the transitions imposed by periodic waveforms, as shown in Figure 1. This result is in agreement with previous findings which demonstrated the accuracy, the consistency, and the efficiency of the RLS algorithm in estimating time-varying versions of Coherence (Möller et al., 2001), Directed Transfer Function, Partial Directed Coherence (Astolfi et al., 2008), and Granger Causality (Hesse et al., 2003; Milde et al., 2010) in simulation studies. Our results demonstrate a significant impact of the forgetting factor on bias, variance, and response time to transitions. Bias increases with higher values of the forgetting factor, while the latter two exhibit an opposite trend. The trends shown in Figure 2 for the investigated indices indicate an intersection within the range of 1 − *c* ∈ [0.97, 0.98], which identifies a potential optimal value for the forgetting factor. Previous studies highlighted the influence of the forgetting factor on the estimation performance in terms of BIAS of estimation (Möller et al., 2001; Hesse et al., 2003; Astolfi et al., 2008; Ciochina et al., 2009; Milde et al., 2010) documenting a suitable range for this parameter between 0.96 and 0.99. This range ensures a proper trade-off between the response time to a transition (which increases for low values of *c*) and the variance of estimate (which increases with high values of *c*). It should be noted that the results obtained in this study may vary depending on several factors, e.g., the number of available data samples for the estimation procedure, the waveform utilized to modify the time-varying autoregressive parameter *a*_1,*n*_, and the number of imposed transitions within the analyzed time window. These factors can potentially influence the estimation performance of the RLS algorithm.

### 5.2 Application to brain-heart interactions

The time-varying estimation of IS was firstly computed on brain signals acquired during a resting condition and then synchronized with the heartbeat to study brain-heart interactions. The grand average distributions over the scalp obtained for the MEAN index (Figure 4) indicate that the information stored in the human brain at rest is primarily localized in the parieto-occipital areas, and this result appears to be independent of the forgetting factor. This finding may suggest a more regular activity of the brain promoted by the activation of the default mode network (DMN). Previous studies in the literature have associated the DMN activity with an increase in EEG power in the alpha and beta frequency bands in parietal and occipital regions (Dirlich et al., 1998; Fox and Raichle, 2007). Additionally, the scalp maps obtained in this study are comparable with those obtained in our previous work, where we computed a local version of IS under stationarity assumption (Barà et al., 2023).

The similarity of the distributions across subjects for the average values of IS within each interval suggests that the physiological phenomena underlying the regularity of neural rhythms are consistent throughout the cardiac cycle and are not influenced by the CFA. In fact, we defined the intervals according to a previous definition (Barà et al., 2023) to mitigate the influence of the CFA, which is known to be prominent in the first and third interval (Dirlich et al., 1997).

The results of the statistical analyses conducted on the MEAN index (Figure 5) reveal significant differences primarily observed in the fronto-temporal and parieto-occipital brain regions. These differences are only evident when 1 − *c* = 0.99. Specifically, the findings suggest that the information stored in the brain signals is higher during interval *I*_2_ than in intervals *I*_1_ and only occasionally *G*. This implies that cardiac activity may impact the predictive information of EEG dynamics, leading to increased regularity and predictability, as measured by the time-varying IS. These results can be related to previous findings suggesting that larger HEPs are localized over the right temporal-parietal regions over the scalp, which play a crucial role in modulating autonomic and behavioral aspects of emotion-related arousal (Luft and Bhattacharya, 2015). However, these comparisons produced a very low effect size (*d* < 0.05) indicating that the study requires a higher number of experimental subjects or merely that the cardiac activity does not have an influence on brain regularity (Sullivan and Feinn, 2012; Barà et al., 2023). The statistical analyses confirm that the results may be affected by the forgetting factor, thus suggesting that the RLS algorithm should be employed with multiple values of forgetting factor, as also confirmed in previous studies (Möller et al., 2001; Astolfi et al., 2008).

The asymmetric trends of the *p*-values shown in Figure 5, obtained by changing the forgetting factor, can be explained through a methodological observation. As demonstrated in the simulation study, the variance of the estimate increases as the value of the forgetting factor decreases. Consequently, the variance of the MEAN index distributions in the different intervals tends to increase, making it more challenging to reject the null hypothesis (no differences between the mean of the IS in the two intervals).

The grand average distributions of the STD index reveal high variability primarily localized over the scalp areas (Figure 6), where the information stored is higher (Figure 4). This finding is consistent with the simulation study results (Figure 1A–C), where higher variance values were associated with higher values of IS in the ON condition when compared to the OFF condition. Furthermore, we note a consistent trend where the STD decreases as the forgetting factor increases. This trend is in line with the results obtained from the simulation studies presented in Figure 2, where an increase in the forgetting factor corresponded to a decrease in the estimation variance.

Another notable finding is the modulation of STD values across the different intervals, indicating that the variability of the information stored in the EEG signals is influenced by the course of time (Figure 6). This result demonstrates that the STD of IS changes depending on whether it is computed globally or within the time windows corresponding to the different phases of the cardiac cycle, thus confirming previous findings reported in (Barà et al., 2023).

The results of the statistical analyses conducted on the STD parameter (Figure 7) reveal a significant decrease in STD values when going from the first to the third interval, regardless of the forgetting factor used for the estimation procedure. This suggests that the cardiac pulse near the R-peak of each heartbeat serves as a trigger and can influence the fluctuation in EEG regularity. Moreover, the gradual reduction in variability over time in the EEG signals indicates a diminishing impact of the heartbeat, which may be associated with a decrease in the perturbation of the IS measure until the occurrence of the next stimulus (Barà et al., 2023). These findings are further supported by the effect size measure, consistently exceeding a value of *d* > 1.5, regardless of the forgetting factor indicating very high statistical significance.

The results presented in this application-oriented context emphasize the significance of EEG regularity variability in exploring the fundamental mechanisms of brain-heart communication. These findings strengthen the efficacy of employing both the mean and variability of time-varying IS, estimated with different forgetting factor values, as a tool for discerning the behavior of interacting physiological systems during transitions across different states.

### 5.3 Further remarks and limitations

The study of the interplay between the brain and the heart could offer a more thorough understanding of the physiological mechanisms that control heart rate, blood pressure, and other cardiovascular processes, as well as helping in the identification of biomarkers that can be predictive of cognitive decline (Ottaviani, 2018) or cardiovascular disease (Doehner et al., 2018). Understanding brain-heart interactions has been identified in the literature as a non-trivial task and the majority of the works debating this topic analyzed the HEPs as a response to the cortical processing of the heartbeat occurring in the brain and reflecting the interaction between the heart and the brain. This potential typically occurs around 200–500 ms after the onset of the R-wave of the ECG signal and can be obtained through the averaging of the EEG traces across multiple trials (Park and Blanke, 2019; Petzschner et al., 2019). However, several studies pointed out how the amplitude and timing of HEPs can be influenced by a variety of factors, including heart rate variability, respiration, emotional state, and cognitive processes, thus leading to contrasting results (Coll et al., 2021). The methodology used to analyze the HEPs is quite different from the time-varying IS and a direct comparison of our results with other studies which used the HEP is difficult to be performed. Indeed, the time-varying approach can uncover the presence of repetitive EEG patterns tied to the heartbeat. However, when using the HEP, the averaged trend of how the cardiac electrical stimulus flows across the scalp can blur the presence of local regularity patterns. Nevertheless, we found a good agreement of the results here presented and other studies in literature using a non-parametric cluster-based permutation technique (Schandry et al., 1986; Park and Blanke, 2019; Coll et al., 2021) or a local version of the IS based on stationarity assumption of the EEG signals (Barà et al., 2023). The former identified the influence of heartbeats on neural activity at the fronto-central electrodes and in a time interval between 300 and 600 ms after the ECG R-peak, confirming the importance of the interval *I*_2_ as the less influenced by the CFA and more related with the emergence of HEPs. The latter studies demonstrated that the heartbeat is capable of evoking alterations in the information processed by the brain activity manifested mainly through the changes in the standard deviation of the local information storage rather than its mean values. Further studies should focus on an exhaustive comparison between the two approaches, highlight similarities and differences, and to provide a comprehensive analysis of the cortical dynamics induced by heartbeat.

Despite the above-reported advantages and potentialities of the proposed novel approach, there are as well some limitations that should be taken into account. First, the use of the Independent Component Analysis (ICA) may lead to remove not only CFA but also important information related to the cortical processing of the heartbeat, being both generated by the same source (Park and Blanke, 2019). One possible solution could be the application of current-source density transformation which allows to minimize EEG signal artifacts while preserving the ability to analyze HEPs (Kayser and Tenke, 2015). Secondly, the analysis here reported should be repeated in the domain of cortical sources to avoid the well-known blurring effect of the EEG and its consequences on the dynamical analysis of EEG data (Anzolin et al., 2019). In addition, it is important to note that the analyses carried out in this study were limited to the resting condition only. To gain a more comprehensive understanding of the HEPs, it is necessary to compare our results to those obtained during experimental conditions where subjects engage in tasks commonly used to study the HEP, such as the heartbeat counting task described in (Schandry et al., 1986), or in the presence of pathological conditions such as depression or nightmare disorder (as investigated in (Terhaar et al., 2012)). Moreover, the statistical comparisons among the various time intervals, in the absence of a second experimental condition, could introduce bias into the analysis. In this study, the time interval during which the CFA is expected to be absent (e.g., *I*_2_) was compared with the other time intervals in which the CFA could potentially be present. Therefore, despite the precautionary application of Independent Component Analysis (ICA), certain statistically significant differences in the MEAN or STD indexes might be attributed to the presence of an artifact.

With regard to the results obtained in this study, a further consideration should be made. Previous works have demonstrated that during the transition from one state to another, the physiological network structure undergoes consistent reorganization (Ivanov et al., 2021). Despite the established association between dominant brain rhythms and emergent physiological states, our understanding of the nature, dynamics, and interaction between different physiological systems across physiological stage transitions remains incomplete. For instance, as shown in (Wang et al., 2019), sleep periods exhibit numerous abrupt transitions among sleep stages and short awakenings, with continuous fluctuations within sleep stages triggering micro-states and brief arousals. Moreover, it has been shown how the variability of heart rate (Sammito et al., 2016), brain activity (Fafrowicz et al., 2019), and their interactions (Candia-Rivera et al., 2022) can be modulated during the day and in response to the executed task. In line with this perspective, heart and brain activities can exhibit fluctuations across various physiological stages in response to the time of day, which, in turn, may affect the obtained results. In the current study, acquisitions were conducted in the early afternoon to ensure stability and reproducibility of experimental conditions for all participants. Therefore, further investigations are needed to explore brain-heart interactions to gain a more comprehensive understanding of the complex relationships between brain activity and heart function during different states of consciousness.

## 6 Conclusion

This study presented a novel approach for measuring time-varying information in a complex system, which can be applied even in non-stationary conditions. The method was validated through simulations and then applied to study brain-heart interactions using real data. The results provided insights into how this approach can track abrupt changes in the information stored in a stochastic process. These changes are primarily reflected in the evolution of the system over time, and in its variance, which becomes higher when there is higher information stored in the system.

The results obtained in the study of brain-heart interactions have shown that, although the average information stored in different phases of the cardiac cycle is comparable, there is a modulation over-time of the local variability of the IS that could be used as a distinctive feature when assessing brain-heart interactions. The proposed method, based on the recursive identification of an AR model, can be exploited as a useful supplementary tool for studying physiological systems where the assumption of stationarity does not hold. However, further analyses are envisaged to more in depth compare the results obtained in this work with those obtained in (Barà et al., 2023) by performing a systematic comparison between the behaviors of the local and the time-varying approaches.

Future developments will aim at testing the proposed novel on different biosignals in the context of network physiology, to provide new tools to analyze over-time the information stored in physiological (Antonacci et al., 2021a; Koutlis et al., 2021) and non-physiological (Antonacci et al., 2021b) complex systems. Moreover, a complete time-varying estimation of the information processing could be provided in a network of multiple interacting dynamical systems in the framework of information dynamics (Faes et al., 2016). As a further step, the recently introduced topology identification via recursive sparse online learning (Zaman et al., 2020) will allow to broaden the approach here introduced with a sparsity constraint (Antonacci et al., 2023), so as to track time-varying information decomposition in network systems.

## Data Availability

The data analyzed in this study is subject to the following licenses/restrictions: The behavioral and physiological raw data can be shared by the corresponding author upon request if data privacy can be guaranteed according to the rules of the European General Data Protection Regulation (EU GDPR). Requests to access these datasets should be directed to a.zaccaro90@gmail.com.
