# Supplementary material for: Time-varying information measures: an adaptive estimation of information storage with application to brain-heart interactions
Source: Front Netw Physiol. 2023 Oct 18;3:1242505. doi: 10.3389/fnetp.2023.1242505 (PMC10619917; doi:10.3389/fnetp.2023.1242505)
Supplement: Supplementary file 1 [file DataSheet1.pdf]

Supplemental Material

1 THE RECURSIVE LEAST-SQUARES (RLS) ALGORITHM

In the following we report the derivation of the RLS solution as described in (Haykin (2002)).
The concept behind the RLS algorithm is the use of an iterative approach to estimate the parameters of a linear system based on its inputs and its outputs. The goal is to minimize the difference between the predicted outputs and the actual outputs, by updating the estimated parameters at each time step n. To achieve this goal it is necessary to minimize the cost function $\mathcal{J}(\mathbf{A}_n)$ with respect to the coefficient matrix $\mathbf{A}_n$. $\mathcal{J}(\mathbf{A}_n)$ is minimized by taking the partial derivatives for all entries of the coefficients vector A_n and setting the results to zero. Then, replacing Z_i with its definition and rearranging the equations leads to:

$$\left(\sum_{i=1}^{n}(1-c)^{n-i}W_iW_i^\top\right)A_n = \sum_{i=1}^{n}(1-c)^{n-i}W_iY_i, \tag{S1}$$

which can be written in compact form as:

$$\mathbf{\Phi}_n^w A_n = \Phi_n^{wy}, \tag{S2}$$

where $\mathbf{\Phi}_n^w \in \mathbb{R}^{p\times p}$ is the time average correlation matrix of the lagged variables and $\Phi_n^{wy} \in \mathbb{R}^{p\times 1}$ is the time average cross-correlation between the the present state and the past states of the process Y. Note that under stationarity assumption ($c = 0$) Eq.(S2) reduces to the well-known Yule-Walker equations (Lütkepohl (2013)) where $\mathbf{\Phi}_n^w = n\mathbf{\Sigma}_{W_n} = n\,\mathbb{E}[W_nW_n^\top]$ allowing to estimate the correlation structure of the process $\forall k = 1, \ldots, p$.
Taking the first term on the left in Eq.(S1), and isolating the term for which $i = n$ from the rest of the summation we obtain a relation for $\mathbf{\Phi}_n^w$ as:

$$\mathbf{\Phi}_n^w = (1-c)\left[\sum_{i=1}^{n-1}(1-c)^{n-1-i}W_iW_i^\top\right] + W_nW_n^\top, \tag{S3}$$

where the expression inside the brackets on the right-hand of Eq.(S3) is the correlation matrix $\mathbf{\Phi}_{n-1}^w$. Thus, we obtain a recursion for the update of the correlation matrix of the past state of Y as follows:

$$\mathbf{\Phi}_n^w = (1-c)\mathbf{\Phi}_{n-1}^w + W_nW_n^\top. \tag{S4}$$

Here, the value of the correlation matrix at the previous state is "corrected" by the product $W_nW_n^\top$. In a similar way we can obtain a recursion for the update of the cross-correlation vector between the past and the present states of Y (i.e., Φ_n^{wy}) by isolating the term $i = n$ in the right side term of Eq.(S1):

$$\Phi_n^{wy} = (1-c)\Phi_{n-1}^{wy} + W_nY_n. \tag{S5}$$

To compute the least-square estimation of A_n in accordance with Eq.(S2) it should be necessary to determine the inverse of the correlation matrix $\mathbf{\Phi}_n^w$, but this operation is quite time consuming especially when the number of AR parameters to be estimated is very high. Moreover, we would like to be able to compute the estimation recursively for $n = 1, \ldots, \infty$. Both of these objectives can be reached through the use of

the *matrix inversion lemma*(Woodbury (1950)), which states that, given $\mathbf{A}$ and $\mathbf{B}$ two positive-definite matrices $M \times M$ related by: $\mathbf{A} = \mathbf{B}^{-1} + \mathbf{C}\mathbf{D}^{-1}\mathbf{C}^{\top}$, where $\mathbf{D}$ is a positive-definite $N \times M$ matrix and $\mathbf{C}$ is an $M \times N$, we can express the inverse of $\mathbf{A}$ as: $\mathbf{A}^{-1} = \mathbf{B} - \mathbf{B}\mathbf{C}(\mathbf{D} + \mathbf{C}^{\top}\mathbf{B}\mathbf{C})^{-1}\mathbf{C}^{\top}\mathbf{B}$. By setting $\mathbf{A} = \mathbf{\Phi}_n^w$, $\mathbf{B}^{-1} = (1-c)\mathbf{\Phi}_{n-1}^w$, $\mathbf{C} = W_n$ and $\mathbf{D} = 1$ with the hypothesis that $\mathbf{\Phi}_n^w$ is non-singular and therefore invertible, it is possible to obtain a recursive equation for the inverse of the correlation matrix by solving the following *Riccati equation* (Hille (1997)):

$$(\mathbf{\Phi}_n^w)^{-1} = (1-c)^{-1}(\mathbf{\Phi}_{n-1}^w)^{-1} - (1-c)^{-1}K_n W_n^{\top}(\mathbf{\Phi}_{n-1}^w)^{-1}, \tag{S6}$$

where $K_n \in \mathbb{R}^{p\times 1}$ is the so-called gain vector defined as the vector of the lagged term W_n, transformed by the inverse of the correlation matrix $\mathbf{\Phi}_n^w$. Directly from the *matrix inversion lemma*, a relationship between the gain vector and the inverse correlation matrix can be obtained as follows:

$$K_n = (\mathbf{\Phi}_n^w)^{-1} W_n. \tag{S7}$$

The matrix $(\mathbf{\Phi}_n^w)^{-1}$ may be also viewed as the covariance matrix of the RLS estimate A_n, normalized with respect to the innovation variance $\sigma^2_{U_n}$ in a multiple linear regression model with stationarity assumption. Now it is possible to define a recursive estimation for the AR vector A_n. To this end, we can rewrite Eq.(S2) by using Eq.(S5) as:

$$A_n = (\mathbf{\Phi}_n^w)^{-1}(1-c)\Phi_{n-1}^{wy} + (\mathbf{\Phi}_n^w)^{-1} W_n Y_n. \tag{S8}$$

Substituting Eq.(S6) for $(\mathbf{\Phi}_n^w)^{-1}$ in the first term only on the right-hand side of Eq.(S8), we obtain:

$$A_n = A_{n-1} - K_n W_n^{\top} A_{n-1} + (\mathbf{\Phi}_n^w)^{-1} W_n Y_n. \tag{S9}$$

Finally, using the fact that $(\mathbf{\Phi}_n^w)^{-1} W_n$ equals the gain vector K_n, we get the desired recursive equation for updating the vector of the AR coefficients:

$$A_n = A_{n-1} + K_n Z_n, \tag{S10}$$

where $Z_n = Y_n - W_n^{\top} A_{n-1} \in \mathbb{R}^{1\times 1}$ is intended as the a-priori estimation error. The inner product $W_n^{\top} A_{n-1}$ represents an estimate of the response Y_n based on the previous least-squares estimate of the AR vector of coefficients that was obtained at time $n-1$. We may view Z_n as a "tentative" value of error before updating the AR coefficients vector.

REFERENCES

Haykin SS. *Adaptive filter theory* (Pearson Education India) (2002).
Lütkepohl H. *Introduction to multiple time series analysis* (Springer Science & Business Media) (2013).
Woodbury MA. *Inverting modified matrices* (Department of Statistics, Princeton University) (1950).
Hille E. *Ordinary differential equations in the complex domain* (Courier Corporation) (1997).
